# Validation of the Scored Patient-Generated Subjective Global Assessment (PG-SGA) in Thai Setting and Association with Nutritional Parameters in Cancer Patients

**DOI:** 10.31557/APJCP.2019.20.4.1249

**Published:** 2019

**Authors:** Nicharach Nitichai, Jongjit Angkatavanich, Nicha Somlaw, Narin Voravud, Chawalit Lertbutsayanukul

**Affiliations:** 1 *Department of Nutrition and Dietetics, Faculty of Allied Health Sciences, Chulalongkorn University,*; 2 *Department of Medicine,*; 3 *Division of Radiation Oncology, Department of Radiology, Faculty of Medicine, Chulalongkorn University and King Chulalongkorn Memorial Hospital, Bangkok, Thailand. *

**Keywords:** Nutritional assessment, nutritional status, scored patient-generated subjective global assessment, malnutrition

## Abstract

**Background:**

The Scored Patient-Generated Subjective Global Assessment (PG-SGA) is a multidimensional tool to assess malnutrition and risk factors. The objectives of this study are to determine the validity of the Thai version of the Scored PG-SGA (Thai PG-SGA) and examine the correlations with selected nutritional parameters.

**Methods:**

This observational analytic study included 195 cancer patients aged greater than 18 years at a university-affiliated hospital in Bangkok, Thailand. All patients were assessed for nutritional status by Thai PG-SGA in comparison to subjective global assessment (SGA). Anthropometry, body composition, and hand grip strength were evaluated**. **

**Results:**

According to PG-SGA global assessment categories, 39% (75) of 195 cancer patients were well nourished, 27% (53) were moderately malnourished and 34% (67) of patients were severely malnourished. Thai PG-SGA had a sensitivity of 99.1% and a specificity of 86.0% at predicting SGA classification. PG-SGA numerical scores were significantly different between well-nourished and malnourished groups (4.2 ± 2.4 Vs 16.3 ± 4.9; p < 0.001). The PG-SGA scores, nutritional status assessed by PG-SGA, and nutritional status assessed by SGA were correlated with weight, % weight loss in one month, body mass index, body fat, and hand grip strength (p < 0.001) respectively.

**Conclusions:**

Thai PG-SGA showed high sensitivity and good specificity in predicting malnutrition in Thai cancer patients. This tool demonstrated the correlations with anthropometric parameters, body composition, and muscle strength.

## Introduction

Oncology patients are at risk of malnutrition throughout the course of the disease and its treatment (Andreyev et al., 1998). The prevalence of malnutrition in cancer patients ranged from 40% to 80% depending on the tumor type, location, stage, and therapy (Ollenschläger et al., 1991; Shike, 1996; Barrera, 2002). Cancer and anti-cancer treatments could adversely affect the patient’s nutritional status, where they interfere with appetite and dietary intake (Capra et al., 2001). All of which leads to malnutrition and may cause an increased risk of complications, reduced response and tolerance of treatment, decreased quality of life, increased healthcare costs, and prolonged hospitalization (Lis et al., 2012; Arends et al., 2017). Therefore, screening, assessment, and monitoring of malnutrition is essential for triaging patients and provided timely intervention to improve clinical outcomes (Bauer et al., 2002). 

The Subjective Global Assessment (SGA) is a well validated tool for assessing nutritional status which has generally been regarded as a standard tool based on the concept of medical history and physical examination (Detsky et al., 1987). It has been used in several clinical settings and has been proven to correlate with clinical variables (anthropometry, biochemistry, clinical and tumor-related characteristics of patients, and quality of life) (Isenring et al., 2003; Li et al., 2011; de Magalhães Cunha et al., 2015). However, this assessment has a limitation of its subjective method in categorizing patient into three categories, which could lead to difficulty to detect small changes in nutritional risks (Barbosa-Silva and Barros, 2006).

The Scored Patient-Generated Subjective Global Assessment (PG-SGA) is a 4-in-1 instrument facilitating proactive screening, assessment, monitoring, and interdisciplinary intervention triage (Jager-Wittenaar and Ottery, 2017). The PG-SGA was developed as a modification of the original SGA and has been widely used as a reference method for nutrition assessment in oncology patients (Bauer et al., 2002; Isenring et al., 2003; Jager-Wittenaar and Ottery, 2017). The scored PG-SGA consists of 2 components. Firstly, the patient-generated component was designed to be completed by patient. It incorporates four boxes on weight history, food intake, nutrition impact symptoms, and activities/function. These components are officially known as the PG-SGA Short Form and to reflect about 80-90% of the total PG-SGA score (Ottery, 1996). Secondly, professional component includes 5 Worksheets addressing scoring the percentage of weight loss, disease and its relation to nutritional requirements, metabolic demand, physical examination, and the global category rating. The professional component was developed to be filled by the healthcare professionals (Ottery, 1996). In addition, the PG-SGA provides a numerical scoring system which helps prioritizing patients to receive urgent interventions matched with their symptoms based on nutritional triage recommendations and monitoring changes in nutritional risks (Soeters et al., 2008; Sealy et al., 2016; Jager-Wittenaar and Ottery, 2017; Ottery, 2015). The advantages of the PG-SGA are not only reducing time for patient interaction and shortening clinic flow but also potentially allowing proactive prevention of malnutrition by identifying and triaging for necessary interventions (Jager-Wittenaar and Ottery, 2017).

Recently, the Thai version of the Scored PG-SGA (Thai PG-SGA) was officially established which included multiple translation processes according to the principles of good practice by the International Society for Pharmacoeconomics and Outcomes Research (ISPOR) (Wild et al., 2005). It was tested in cancer patients and healthcare professionals with the supervision of copyright holder and an international expert on translation and cultural adaptation of the PG-SGA. The results showed it has conceptual equivalence to the original English PG-SGA and considered easy to use and comprehensible by cancer patients and healthcare professionals (Nitichai et al., 2018). Utilization of this tool in each own country’s language could better reflect nutrition status of cancer patients as well as promote meta-analysis and inter-country comparison of nutrition status in cancer patients since the Scored PG-SGA was translated and culturally adapted to several languages (Jager-Wittenaar and Ottery, 2017). Nonetheless, at present, the validity of Thai PG-SGA and its association with important clinical parameters has not been assessed.

The aims of this study were to validate Thai PG-SGA by comparing with SGA in categorizing nutritional status of cancer patients and evaluate the association of this tool with anthropometry, body composition, and hand grip strength. 

## Materials and Methods

This study was a cross-sectional design to validate the Thai version of the Scored PG-SGA with the SGA in assessing nutritional status in cancer patients and concurrently analyze the relationship of the assessment outcomes with body weight, body mass index, percentage of weight loss, percentage of body fat, muscle mass, and hand grip strength.

*Participants*

Cancer patients at the outpatient and the inpatient departments of Division of Therapeutic Radiation and Oncology, King Chulalongkorn Memorial Hospital, Bangkok, Thailand were recruited into the study by convenience sampling between February and April 2017. Eligibility criteria were as follows: age greater than 18 years, having anticancer treatment (chemotherapy and/or radiotherapy and/or surgery), and agreed to participate in the study. Patients having physical limitation or cognitive impairments, being pregnant, or being unable to read and write in Thai, were excluded.

The study was approved by the Institutional Review Board of the Faculty of Medicine, Chulalongkorn University (COA No. 603/2016, IRB No. 259/59). All eligible participants were informed about the study protocol and gave their written consent before participating in the study.

*Nutritional assessment*

The nutritional status of all cancer patients was assessed using Thai PG-SGA (Nitichai et al., 2018) and SGA (Detsky et al. 1987) by a trained dietitian experienced in using both tools. In the evaluation process, the trained dietitian applies strict criteria to the standard protocols and making measurements with great care in categorizing nutritional status by PG-SGA and SGA. The details of category rating and numerical scoring system of PG-SGA and SGA were addressed by Jager-Wittenaar (2017) and Detsky (1987) respectively. Permission by copyright owner of PG-SGA and SGA were granted. Thai PG-SGA is now available at www.pt-global.org. Each patient was either classified as well nourished (category A), moderately malnourished or suspected of being malnourished (category B), or severely malnourished (category C). The total score of PG-SGA were the sum of scores from patient-generated component and professional component, where the higher score indicating higher severity of malnutrition. The score of 0-1 suggests no intervention required, 2-3, educating patient and family is recommended, 4-8, requiring intervention as indicated by symptoms, and the score of 9 or more implying critical need for intervention. The score was also affected by age, diagnosis, and stages of cancer. These factors were retrieved from medical records.

*Anthropometric assessment*

Body weight (kg), body fat (%) and muscle mass (kg) were measured by a bioelectrical impedance analysis (BIA) machine (Tanita™ Body Composition Monitor, BC 545 model). The body weight was recorded in kilogram to the nearest 0.1 kg. A stadiometer was used for measuring height and recorded in centimeter to the nearest 0.1 cm. Body mass index was calculated as body weight in kg per height in m^2^ (kg/m^2^). The anthropometric measurement was evaluated by the same researcher throughout the study.

*Functional assessment*

Hand grip strength (HGS) was measured using a mechanical grip dynamometer (T.K.K. 5001 Grip A). All study patients performed the test while sitting comfortably with the arm by their side of the body and the elbow flex at 90^o^. The test was administered with the dominant hand. The patients squeezed the handgrip dynamometer as hard as possible for three consecutive measurements with a 30 seconds rest period between each squeeze to allow for optimal recovery. The mean of the three trials were used as the output measure for the test. All measurements were performed by the same investigator to avoid inter-observer variation. 

*Statistical analysis*

All data were analyzed using the Statistical Package for the Social Sciences (SPSS) version 22.0 for Windows. Descriptive statistics were reported as frequency, percentage, mean ± standard deviation (SD) or median and interquartile range. A contingency table was used to determine the sensitivity, specificity, and predictive value of the Thai version PG-SGA compared to SGA. Data between well-nourished and malnourished group were compared. The continuous clinical variables with normal distribution (body weight, BMI, % weight loss, body fat, handgrip strength, PG-SGA scores) and skewed distribution (age and muscle mass) were analyzed by independent t-test and Mann-Whitney U test respectively. Chi-square tests were used for categorical clinical variables. Correlations between PG-SGA scores, nutritional status classified by PG-SGA/SGA and clinical variables were reported as the Pearson correlation coefficient and Spearman’s rho correlation. The strength of the correlation for the absolute value of r: 0.00 - 0.19 “very weak”, 0.20 - 0.39 “weak”, 0.40 - 0.59 “moderate”, 0.60 - 0.79 “strong”, and 0.80-1.00 “very strong”. A p-value < 0.05 was considered statistically significant.

## Results

*Clinical characteristics and prevalence of malnutrition of study participants*

A total of 195 cancer patients were recruited. There were more females (63%) than males (37%), with median age of 58 years old. The majority of patients were diagnosed with breast cancer, followed by head and neck cancer. According to Thai PG-SGA, 75 (39%) of patients were well-nourished (PG-SGA A), 53 (27%) were moderately/suspected malnutrition (PG-SGA B) and 67 (34%) were severely malnutrition (PG-SGA C). Detailed patient characteristics classified by PG-SGA categories are shown in Table 1.

*Comparison of clinical variables between well-nourished and malnourished patients*

Malnourished cancer patients had significantly higher average percentage of weight loss in one month and lower handgrip strength compared to those who classified as well-nourished (p <0.001). On the contrary, actual body weight, BMI, % body fat and muscle mass did not differ between well-nourished and malnourished patients. Moreover, the mean PG-SGA scores were significantly higher in malnourished group (16.3 ± 4.9) than well-nourished group (4.2 ± 2.4) (Table 1).

*Validity of the Thai version of the Scored PG-SGA*

The ability of the Thai version of the Scored PG-SGA to predict SGA is shown in Table 2. The contingency table showed 108 (55.38%) of 195 cancer patients were correctly classified as malnourished by PG-SGA (true positive, TP) and 74 (37.95%) of patients were correctly identified as well-nourished (true negative, TN). Moreover, there were few patients misclassified as malnourished (false positive, FP) and well-nourished patients (false negative, FN). The Thai version of the Scored PG-SGA had a sensitivity of 99% and a specificity of 86%. The positive predictive value and negative predictive value were 90.0% and 98.7% respectively. The accuracy of the Thai version of the Scored PG-SGA was 93.3%.

*Association between PG-SGA scores, nutritional status and clinical variables*

Table 3 summarizes the correlations between PG-SGA scores, nutritional status classified by PG-SGA/SGA and clinical variables. There was a strong and significant positive correlation between the nutritional status assessed by the PG-SGA and SGA (r= 0.87, p < 0.001). A strong and significant positive correlation also found between PG-SGA numerical scores and nutritional status assessed by PG-SGA (r= 0.84, p < 0.001) and SGA (r= 0.82, p < 0.001). 

For the anthropometric assessment, percentage of weight loss in one month had positive correlation with PG-SGA scores (r=0.66, p < 0.001), nutritional status assessed by PG-SGA and SGA (r=0.54, 0.50, p < 0.001). In contrast, actual body weight, BMI, and % body fat showed inverse correlation with PG-SGA scores, nutritional status assessed by PG-SGA and SGA. In addition, functional assessment by handgrip strength showed negative correlation with PG-SGA scores, nutritional status identified by PG-SGA and SGA.

## Discussion

This was the first study to determine the validity of the official Thai translation and cultural adaptation PG-SGA in predicting malnutrition as compared to SGA in terms of sensitivity and specificity. Thai PG-SGA was shown to be valid at identifying malnutrition among Thai cancer patients. The results are consistent with Bauer et al., (2002) who stated that the Scored PG-SGA had 98% sensitivity and 82% specificity when compared to SGA. This may be explained by the similarity of study design which was conducted in oncology setting and experience of dietitian in using the tool. When comparing with SGA as the gold standard, PG-SGA seems to have a lower specificity may be due to the scores obtained from the nutrition impact symptoms and other factors (box 3). In this part, any symptoms affecting eating that patient reports get scored and all points are additive (maximum 24 points in this section) (Jager-Wittenaar and Ottery, 2017). These extensive range of symptoms provided by PG-SGA may identify more patients at risk of malnutrition. Being able to identify nutrition impact symptoms at the early stage could be beneficial for proactively preventing malnutrition allowing timely intervention during cancer and anti-cancer treatments. Another possibility for the difference between PG-SGA and SGA is the contribution of the assessment of weight change where weight information in PG-SGA is addressed along a continuum – 6 months (chronic), 1 month (intermediate), and past two weeks (acute) change but that of SGA change is assessed at 2 time-points, only in the past 6 months and 2 weeks.

The prevalence of malnutrition assessed by Thai PG-SGA was high (62%) probably due to the severity of cancer in majority of malnourished patients and inclusion of hospitalized cancer patients. The study findings indicated that cancer patients with advanced stage had a higher prevalence of malnutrition which was comparable to the figure reported by Bauer et al., (2002) in which 76% of cancer patients were found to be malnourished as classified by PG-SGA. 

In this study, mean PG-SGA scores were significantly higher in malnourished compared to well-nourished patients which was consistent with previous studies (Bauer et al., 2002; Laky et al., 2008; Gabrielson et al., 2013). The PG-SGA numerical scores showed positive correlation with nutritional status assessed by PG-SGA and SGA, in the anticipated direction. 

The percentage of weight loss in one month was significantly higher in malnourished than well-nourished group. It also had significantly positive correlation with PG-SGA numerical scores, nutritional status assessed by PG-SGA and SGA. This finding was consistent with Bauer et al., (2002) which reported significant correlation between PG-SGA scores and % weight loss in 6 months (r= 0.31, p = 0.012). Even though, the time interval of % weight loss of the present study is shorter, the results follow the same direction. In this study, actual body weight and BMI were not significantly different between well-nourished and malnourished patients. This was also reported by Gabrielson et al., (2013) that body weight was not significantly different between group (p = 0.218). Another study conducted in 148 lung cancer patients also showed the same trend that body weight and BMI alone failed to identify malnutrition in the patients (Li et al., 2011). Actual body weight and BMI were negatively correlated with PG-SGA scores, nutritional status assessed by PG-SGA and SGA as expected. However, the findings indicated that BMI and body weight alone may have limitations in predicting malnutrition since malnourished cancer patients may have normal or overweight BMI range. Therefore, malnutrition can happen at any BMI while, in some cases, body fat could mask loss of lean body mass (Bauer et al., 2002; White et al., 2012).

Body composition analysis by BIA showed no significant difference of % body fat and muscle mass between well-nourished and malnourished patients. Similar results were found in study by Laky et al., (2008) with no significant difference of fat mass and fat-free mass between well-nourished and malnourished gynecologic cancer patients. In addition, % body fat was found negatively correlated with PG-SGA scores, nutritional status classified by PG-SGA and SGA. Although the correlation of PG-SGA scores, and nutritional status identified by PG-SGA/SGA with muscle mass did not show statistical significance, the direction of the association demonstrated that the higher the PG-SGA scores and the degree of malnutrition, the lower the muscle mass. 

**Table 1:** Clinical Variables for Cancer Patients as Classified by PG-SGA Global Assessment Categories

Clinical variables	PG-SGA Global Assessment Categories			P-value
	Well-nourished patients	Malnourished patients	All patients (n=195)	
	(PG-SGA A)	(PG-SGA B+C)		
Age (years)	57	58	58	0.776 ^a^
	(47.0 - 65.0)	(47.0 - 64.8)	(47.0 - 65.0)	
Gender (Male/Female)	75 (17/58)	120 (56/64)	195 (73/122)	0.001*, ^b^
Primary tumor localization; n (%)				< 0.001*, b
Breast	35 (67.3)	17 (32.7)	52 (26.7)	
Head and neck	16 (33.3)	32 (66.7)	48 (24.6)	
Gynecologic	6 (24.0)	19 (76.0)	25 (12.8)	
Digestive/ gastrointestinal	3 (12.5)	21 (87.5)	24 (12.3)	
Respiratory	2 (18.2)	9 (81.8)	11 (5.6)	
Neurologic	1 (11.1)	8 (88.9)	9 (4.6)	
Genitourinary	4 (50.0)	4 (50.0)	8 (4.1)	
Musculoskeletal	2 (33.3)	4 (66.7)	6 (3.1)	
Endocrine	5 (83.3)	1 (16.7)	6 (3.1)	
Others ‡	1 (20.0)	4 (80.0)	5 (2.6)	
Unknown primary organ	0 (0.0)	1 (100.0)	1 (0.5)	
Stage of cancer; n (%)				0.003 *, ^b^
Stage 0	0 (0.0)	1 (100.0)	1 (0.5)	
Stage I	11 (78.6)	3 (21.4)	14 (7.2)	
Stage II	11 (47.8)	12 (52.2)	23 (11.8)	
Stage III	16 (44.4)	20 (55.6)	36 (18.5)	
Stage IV	20 (26.7)	55 (73.3)	75 (38.5)	
Unknown stage	17 (37.0)	29 (63.0)	46 (23.6)	
Actual body weight (kg)	60.6 ± 13.9	52.4 ± 10.6	55.6 ± 12.5	0.226 ^c^
BMI (kg/m^2^)	24.4 ± 4.7	20.0 ± 3.8	21.7 ± 4.7	0.230 ^c^
Weight loss in 1 month (%)	0.5 ± 1.6	6.0 ± 8.0	3.9 ± 6.9	< 0.001*, ^c^
Body fat (%)	30.8 ± 9.3	20.7 ± 10.5	24.8 ±11.2	0.544 ^c^
Muscle mass (kg)	36.5 (33.2 - 41.9)	36.6 (32.9 - 41.8)	36.6 (33.0 - 41.8)	0.728 ^a^
Hand grip strength (kg)	23.8 ± 6.9	20.0 ± 8.1	21.5 ± 7.9	< 0.001*, ^c^
PG-SGA numerical scores	4.2 ± 2.4	16.3 ± 4.9	11.7 ± 7.2	< 0.001*, ^c^

Data are presented as mean ± standard deviation number, or median (25th and 75th percentile in brackets); PG-SGA: Patient-Generated Subjective Global Assessment, BMI: body mass index; a, p-values are for comparisons between well-nourished and malnourished patients using Mann-Whitney-U test; b, p-values are for comparisons between well-nourished and malnourished patients using Chi-square; c, p-values are for comparisons between well-nourished and malnourished patients using Independent t-test; ‡, Neck lymph node/ Thymus gland/ Skin cancer;

*, p value < 0.05.

**Table 2:** Classification of Nutritional Status in 195 Patients with Cancer According to the Thai Patient-Generated Subjective Global Assessment (Thai PG-SGA) and Subjective Global Assessment (SGA)

Thai PG-SGA	SGA		
	Malnourished (SGA B+C)	Well-nourished (SGA A)	Total
Malnourished	True positive 108 (55.38%)	False positive 12 (6.15%)	All positive 120
Non-malnourished	False negative 1 (0.51%)	True negative 74 (37.95%)	All negative 75
Total	All with malnourished 109	All with well-nourished 86	Total patients 195

**Table 3:** Correlations between Measured Clinical Variables, PG-SGA Scores, and Nutritional Status Classified by PG-SGA/SGA

Clinical variables	PG-SGA numerical scores		Nutritional status classified by PG-SGA Category		Nutritional status classified by SGA Classification	
	r	p-value	r	p-value	r	p-value
PG-SGA numerical scores	1	-				
Nutritional status by PG-SGA	0.84	< 0.001^b^	1	-		
Nutritional status by SGA	0.82	< 0.001^b^	0.87	< 0.001 ^b^	1	-
Actual body weight (kg)	-0.34	< 0.001^a^	-0.36	< 0.001 ^b^	-0.3	< 0.001 ^b^
BMI (kg/m^2^)	-0.45	< 0.001^a^	-0.48	< 0.001 ^b^	-0.44	< 0.001 ^b^
% Weight loss in 1 month (%)	0.66	< 0.001^a^	0.54	< 0.001 ^b^	0.5	< 0.001 ^b^
Body fat (%)	-0.43	< 0.001^a^	-0.46	< 0.001 ^b^	-0.44	< 0.001 ^b^
Muscle mass (kg)	-0.09	0.237 ^a^	-0.09	0.224 ^b^	-0.03	0.729 ^b^
Hand grip strength (kg)	-0.34	< 0.001^a^	-0.36	< 0.001 ^b^	-0.3	< 0.001 ^b^

a, Pearson’s correlation; b: Spearman's rho correlation

Regarding functional assessment of upper extremities by hand grip dynamometer, it was found that well-nourished had significantly higher average HGS than malnourished patients. This is consistent with the finding in a prospective cross-sectional study conducted in 189 cancer patients to examine muscle strength that well-nourished patients had significantly higher HGS than malnourished ones (30.4±10.4 vs. 22.9±11.1 kg) (Norman et al., 2010). The correlation analysis showed that HGS had significantly negative correlation with nutritional status assessed by both tools and PG-SGA scores. These data would suggest that the lower the hand grip strength, the higher the prevalence of malnutrition and PG-SGA scores. Similar trend was found in several previous studies. The study by Ozorio et al., (2017) in 101 gastrointestinal cancer patients found inverse correlation between HGS and PG-SGA (r= -0.52). Another study by Flood (2014) conducted in 217 hospital patients reported negative correlation between HGS and PG-SGA scores (r = -0.292, p < 0.001). HGS has advantages i.e. easy, non-invasive and most feasible bed side method, as well as an indicator of muscle function (Norman et al., 2011). Until now, there is no reliable and validate cutoff value of HGS available for cancer patients and there is no standard protocol for HGS assessment (Norman et al., 2011). This might limit the generalizability of study results since there are different methods in assessing HGS in terms of techniques used and types of instrument. In addition to malnutrition, there are various factors related to muscle weakness such as disease severity, comorbidities and medical treatments (Norman et al., 2011).

From these findings, the correlations between clinical variables and nutritional status assessed by both tools ranged from strong to weak correlations. The results revealed strong correlation between percentage of weight loss in one month and PG-SGA numerical scores and moderate correlation with nutritional status classified by PG-SGA/SGA. Therefore, percentage of weight loss in one month is shown to be significant clinical parameter, a better indicator than other clinical variables. The results also suggested that nutritional status could not be determined by using any single clinical parameter alone because each parameter has different limitation in nutrition assessment. Therefore, data collection from a variety of domains are necessary for nutrition assessment to determine appropriate diagnosis of malnutrition (Jensen et al., 2012). The systematic review showed that PG-SGA could serve as a nutritional assessment tool as it covers all components of the definitions of malnutrition as published by European Society for Clinical Nutrition and Metabolism (ESPEN) and the American Society for Parenteral and Enteral Nutrition (ASPEN) (Sealy et al., 2016). It also has several advantages as a nutritional instrument in comparison to SGA in terms of numerical scoring system rather than category. In addition, it provides extensive range of nutritional impact symptoms which often experienced by oncology patients (Bauer et al., 2002; Jager-Wittenaar and Ottery, 2017).

The strength of this study is that some clinical parameters were evaluated by anthropometric and functional assessment. In addition, there were various kinds of cancer patients with all cancer staging and treatment from both outpatient and inpatient department enrolled to the study.

The principal limitation of this study is the acquiring of participants by convenience sampling and the exclusion of cancer patients who had cognitive impairments and physical limitation that prevented them from completing the Scored PG-SGA. This limitation may influence the study results and limit the generalizability. Another potential limitation of this study is that the nutritional assessment was evaluated by trained dietitian only which may affect to the results. However, it avoids inter-rater variability. Further study is needed to assess interobserver reliability or reproducibility of Thai PG-SGA evaluation among health professionals by a standard method such as a test-retest model. More could be explored for validate the use of Thai PG-SGA in specific type of cancer as well as different treatment regimens. Currently, the Thai PG-SGA has only paper-based version. Development of an application-based tool may yield a better result in terms of reducing time spent, accuracy of PG-SGA score calculation, and convenience of data collection.

In conclusion, the results of this study suggest that Thai PG-SGA is a valid nutritional instrument in identifying malnutrition among cancer patients. This tool could serve as a suitable assessment method for cancer patients in Thailand. The nutritional status assessed by Thai PG-SGA is well correlated with percentage of weight loss in one month. The other clinical variables, while having a weak to moderate correlation, were also partially contribute to nutrition diagnosis. Therefore, the multidimensional tools which include assessment of various key clinical variables could be used to identify appropriate nutritional status. 

## Funding Statement

This study was supported by the 90^th^ Anniversary of Chulalongkorn University Fund (Ratchadaphiseksomphot Endowment Fund)

## Statement conflict of Interest

No conflict of interest in this study.
